# Dynamics of parkinsonian oscillations mediated by transmission delays in a mean-field model of the basal ganglia

**DOI:** 10.3389/fncel.2024.1344149

**Published:** 2024-03-14

**Authors:** Atefeh Asadi, Mojtaba Madadi Asl, Alireza Valizadeh, Matjaž Perc

**Affiliations:** ^1^Department of Neurology, University Hospital Würzburg, Würzburg, Germany; ^2^School of Biological Sciences, Institute for Research in Fundamental Sciences (IPM), Tehran, Iran; ^3^Pasargad Institute for Advanced Innovative Solutions (PIAIS), Tehran, Iran; ^4^Department of Physics, Institute for Advanced Studies in Basic Sciences (IASBS), Zanjan, Iran; ^5^Faculty of Natural Sciences and Mathematics, University of Maribor, Maribor, Slovenia; ^6^Department of Medical Research, China Medical University Hospital, China Medical University, Taichung, Taiwan; ^7^Community Healthcare Center Dr. Adolf Drolc Maribor, Maribor, Slovenia; ^8^Complexity Science Hub Vienna, Vienna, Austria; ^9^Department of Physics, Kyung Hee University, Seoul, Republic of Korea

**Keywords:** transmission delay, brain oscillations, mean-field model, Parkinson's disease, firing rate, basal ganglia

## Abstract

**Introduction:**

Neural interactions in the brain are affected by transmission delays which may critically alter signal propagation across different brain regions in both normal and pathological conditions. The effect of interaction delays on the dynamics of the generic neural networks has been extensively studied by theoretical and computational models. However, the role of transmission delays in the development of pathological oscillatory dynamics in the basal ganglia (BG) in Parkinson's disease (PD) is overlooked.

**Methods:**

Here, we investigate the effect of transmission delays on the discharge rate and oscillatory power of the BG networks in control (normal) and PD states by using a Wilson-Cowan (WC) mean-field firing rate model. We also explore how transmission delays affect the response of the BG to cortical stimuli in control and PD conditions.

**Results:**

Our results show that the BG oscillatory response to cortical stimulation in control condition is robust against the changes in the inter-population delays and merely depends on the phase of stimulation with respect to cortical activity. In PD condition, however, transmission delays crucially contribute to the emergence of abnormal alpha (8–13 Hz) and beta band (13–30 Hz) oscillations, suggesting that delays play an important role in abnormal rhythmogenesis in the parkinsonian BG.

**Discussion:**

Our findings indicate that in addition to the strength of connections within and between the BG nuclei, oscillatory dynamics of the parkinsonian BG may also be influenced by inter-population transmission delays. Moreover, phase-specificity of the BG response to cortical stimulation may provide further insight into the potential role of delays in the computational optimization of phase-specific brain stimulation therapies.

## 1 Introduction

Parkinson's disease (PD) is a multiscale movement-related disorder, affecting the entire cortico-basal ganglia (BG)-thalamo-cortical (CBGTC) circuits involved in motor control at the level of single neurons as well as network interactions (McGregor and Nelson, 2019; Scherer et al., 2022). Degeneration of dopaminergic neurons in the substantia nigra pars compacta (SNc) triggers a cascade of maladaptive or compensatory changes during parkinsonism which alter the dynamics of the striatum and, consequently, the thalamus and cortex (Madadi Asl et al., 2022b). Changes in cortico-striatal dynamics following dopamine (DA) loss massively modulates downstream projections (Day et al., 2006; Fan et al., 2012; Miguelez et al., 2012). Motor symptoms of PD are traditionally viewed as the result of alterations in discharge rates of different CBGTC circuits (Wichmann and DeLong, 2006; Galvan et al., 2015; Asadi et al., 2022). For instance, elevated discharge rates of the subthalamic nucleus (STN) (Mallet et al., 2008b) and globus pallidus internus (GPi) (Kita and Kita, 2011a), and reduced activity of the globus pallidus externus (GPe) (Mallet et al., 2008a) were often observed experimentally. However, discharge rate changes, alone, are not sufficient to explain experimental findings. Rather, changes in the power spectrum of the BG oscillations are pathophysiologically important as well. For example, neural correlates of PD include changes of oscillatory brain activity in alpha (8–13 Hz) and beta band (13–30 Hz) in the electroencephalogram (EEG) and magnetoencephalogram (MEG) (Soikkeli et al., 1991; Bosboom et al., 2006; Stoffers et al., 2007) as well as enhanced synchronized oscillations at beta frequencies in the BG, thalamus, and cortex (Gatev et al., 2006; Galvan et al., 2015).

Experimental findings during the past years refined our understanding of the BG circuitry, e.g., due to the recognition of distinct subpopulations of neurons within the striatum and GPe as well as additional projections (Gertler et al., 2008; Planert et al., 2010; Mallet et al., 2012; Mastro et al., 2014). Any attempt to model the role of these subpopulations and projections in the BG dynamics rapidly runs into difficulties either due to lack of knowledge or computational cost of detailed, biologically-realistic models. In this context, simple models may ignore some details but they are still able to make reasonable predictions. For instance, mean-field models of the BG were able to explain firing rate changes during parkinsonism (van Albada and Robinson, 2009) as well as oscillations and spectral changes in the EEG recordings (van Albada et al., 2009). The consistency of these predictions were later improved by reducing complex BG dynamics to a lower dimensional space of sensibly chosen dynamical features enabling more accurate parameter estimation for the firing rate models of PD (Bahuguna et al., 2017).

Altered response of the BG to transient cortical stimuli is one of the dynamical changes due to DA loss in PD (Kita and Kita, 2011a; Chiken et al., 2021). More precisely, in control (normal) condition the response of the BG to cortical stimulation is triphasic composed of early excitation, inhibition, and late excitation in the GPi and GPe (Jaeger and Kita, 2011; Chiken et al., 2021). In PD, this response is altered such that cortical stimulation-induced inhibition in the GPi is reduced, whereas late excitation in the GPe is elongated (Jaeger and Kita, 2011; Chiken et al., 2021). Since motor commands originate in the cortex, a number of computational studies employed mean-field models to shed light on complex, network-level changes in the BG that may be responsible for pathological cortical information flow in PD (van Albada and Robinson, 2009; van Albada et al., 2009; Kerr et al., 2013; Bahuguna et al., 2017). However, signal transmission across the brain is not instantaneous. Rather, neural interactions and, consequently, the emergent dynamics are significantly affected by transmission delays (Barardi et al., 2014; Madadi Asl et al., 2017, 2018b).

During the past years, a number of studies took into account the role of transmission delays in generating pathological beta oscillations in computational models of the parkinsonian BG (Humphries et al., 2006; Nevado-Holgado et al., 2010; Dovzhenok and Rubchinsky, 2012; Pavlides et al., 2012; Pasillas-Lépine, 2013). For example, Nevado-Holgado et al. (2010) as well as Pasillas-Lépine (2013) developed a mean-field model of the STN-GPe network and showed that transmission delays between the STN and GPe neurons are necessary for the emergence of beta oscillations in the model. In a conductance-based model of the STN-GPe circuit, Dovzhenok and Rubchinsky (2012) introduced two delay units representing synaptic and conductance delays in the CBGTC loop and showed that delayed feedback loop may be essential for tremor-like oscillations in the model. In a mean-field model of the BG, Pavlides et al. (2015) showed that the frequency of STN oscillations mostly depends on the range of delays assigned to the pathway within which the oscillations were driven, i.e., the frequency is lowest (highest) when oscillations are driven by the long (short) delayed loop.

**Figure 1:**
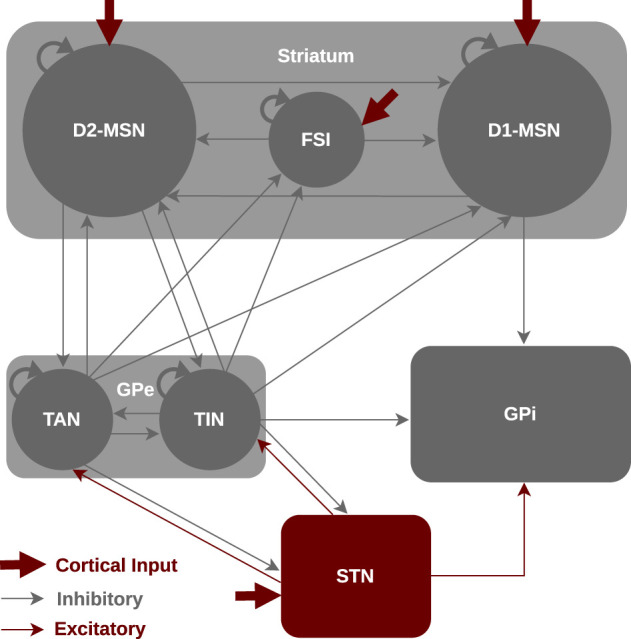
Schematic illustration for the mean-field model of the BG circuitry. The BG model consists of seven nuclei including D1-MSNs, D2-MSNs, FSIs, GPe-TANs, GPe-TINs, STN and GPi interacting via excitatory (red) or inhibitory (gray) connections. The classical functional organization of the BG includes the direct pathway (cortex → striatal D1-MSN → GPi), indirect pathway (cortex → striatal D2-MSN → GPe ⇌ STN → GPi) and hyperdirect pathway (cortex → STN → GPi).

Still, to what extent delays may affect signal transmission in the BG pathways is not well understood. In this study, we investigate how introducing transmission delays into the communication between BG nuclei affects information transmission in the BG driven by cortical stimuli. The goal of the study is to address the following questions: (i) How do changes in inter-population transmission delays alter the discharge rate of the BG nuclei in the control and PD states? (ii) How do phase-specific cortical stimuli compensate for delayed information transmission in the normal and parkinsonian BG? And (iii) on a macroscopic scale, how do these changes influence oscillations and their spectral properties in the control and PD states? To this end, we considered a Wilson-Cowan (WC) model as a mean-field firing rate model with physiologically plausible parameter estimates that can predict discharge rate and power spectrum changes in relation to the control and PD conditions. Our BG model consists of striatum, GPe, STN and GPi, as schematically shown in Figure 1. The model incorporates three distinct subpoplations within the striatum, namely, D1-receptor-expressing medium spiny neurons (D1-MSNs), D2-receptor-expressing medium spiny neurons (D2-MSNs) and fast-spiking interneurons (FSIs) (Gertler et al., 2008; Planert et al., 2010) as well as two distinct subpoplations within the GPe, i.e., tonically active neurons (TANs) and tonically inactive neurons (TINs) (Mallet et al., 2012; Mastro et al., 2014). The advantage of a mean-field model is that it can predict large-scale properties of neural populations with few parameters and evaluate their dependence on inter-population connection strengths and, furthermore, directly assess rate and spectral changes due to transmission delays.

Specifically, we investigated the effect of inter-population transmission delays on the dynamics of BG oscillatory activity. Our results show that changes of delays in the indirect pathway may change the firing pattern of oscillations while the mean firing rates of the BG nuclei are mainly preserved. Moreover, the power of alpha and beta oscillatory activity have the potential to be significantly enhanced in the PD condition in comparison to the control condition due to the presence of inter-population delays. This suggests that delays play a modifying role in generating abnormal oscillations in the parkinsonian BG. Furthermore, we investigated the response of the networks to a transient cortical stimulus and demonstrated that in the control condition the BG oscillatory dynamics is fairly robust against changes of inter-population delays, whereas in the PD condition delays and the phase of cortical stimulation may jointly determine the response of the network to cortical stimuli. Our findings highlight the role of inter-population transmission delays in the emergence of abnormal oscillations in PD. The delay-dependency and phase-specificity of the response of the parkinsonian BG to cortical stimulation may be potentially useful for therapeutic purposes.

## 2 Methods

### 2.1 Mean-field model of the BG

As it is schematically shown in Figure 1, we considered a WC model of the mean activity of different BG nuclei including D1-MSNs, D2-MSNs, FSIs, GPe-TANs, GPe-TINs, STN, and GPi. The model was implemented in MATLAB. Specifically, the striatum is organized into three subpopulations of neurons (Gertler et al., 2008; Planert et al., 2010), i.e., D1-MSNs, D2-MSNs, and FSIs. The D1-MSNs project to the GPi and D2-MSNs project to the GPe cells, whereas the FSIs only project to the neurons within the striatum. The GPe incorporates two subpopulations of neurons (Mallet et al., 2012; Mastro et al., 2014), i.e., TANs and TINs. Both GPe subpopulations project to the striatum as well as STN, whereas TINs also project to the GPi. The TANs fire in-phase with cortical slow-wave activity (SWA) and beta activity in parkinsonian state. The TINs, on the other hand, fire anti-phase with cortical SWA and beta activity in parkinsonian state. The STN receives input from the cortex and GPe, and projects to the GPe as well as GPi. Experiments on the synaptic properties of the STN suggested an absence of intrinsic connectivity (Steiner et al., 2019), therefore, we did not assume an intrinsic input for the STN (i.e., STN → STN projection) in our model.

The dynamics of the system obeys the following differential equation (Bahuguna et al., 2017):


(1) $$\begin{array}{l} \tau \dot{Y}=-Y+S\left(A\cdot Y+B\cdot \lambda_{\text{CTX}}\right), \end{array}$$

where τ = 15*ms* is the time constant and λ_CTX_ = *I*_CTX_+*I*_stim_ represents the sum of the firing rates of cortical input *I*_CTX_ and cortical stimulation current *I*_stim_ (see below).

Moreover, Y(t) is the population firing rate vector and *S*(*x*) is a sigmoidal activation function given by the following relations, respectively:


(2) $$\begin{array}{l} Y\left(t\right)={\left[\begin{array}{c} \lambda_{\text{D}1\left(t\right)},\lambda_{\text{D}2\left(t\right)},\lambda_{\text{FSI}\left(t\right)},\lambda_{\text{TAN}\left(t\right)},\lambda_{\text{TIN}\left(t\right)},\lambda_{\text{STN}\left(t\right)},\lambda_{\text{GPi}\left(t\right)} \end{array}\right]}^{T}, \end{array}$$

(3) $$S(x(t-d))=\frac{\lambda_{\text{max}}}{\text{1}+e^{-a(x(t-d)-\theta )}},$$

where *d* represents the delay in the transmission of signals between populations. The values of θ and λ_max_ shown in Table 1 were tuned to mimic realistic instantaneous firing rates under different input conditions (Bahuguna et al., 2017). For each nucleus, the parameters θ and λ_max_ were fixed for both the control and PD conditions.

**Table 1:** Model parameters for the sigmoidal activation function *S*(*x*) in Equation (3) (Bahuguna et al., 2017).

**Population**	**θ**	**λ_max_**
D1-MSN	0.1	65
D2-MSN	0.1	65
FSI	0.1	80
TAN	0.4	75
TIN	0.4	125
STN	0.4	500
GPi	0.1	250

The temporal derivative of the firing rate vector is given by:


(4) $$\begin{array}{l} \dot{Y}=\frac{dY}{dt}={\left[\begin{array}{c} \frac{d\lambda_{\text{D}1}}{dt},\frac{d\lambda_{\text{D}2}}{dt},\frac{d\lambda_{\text{FSI}}}{dt},\frac{d\lambda_{\text{TAN}}}{dt},\frac{d\lambda_{\text{TIN}}}{dt},\frac{d\lambda_{\text{STN}}}{dt},\frac{d\lambda_{\text{GPi}}}{dt} \end{array}\right]}^{T}, \end{array}$$

where the letter *T* stands for the matrix transpose.

The coupling matrices A and B represent the recurrent and input connection matrices, respectively. An element *J*_*i, j*_ of A and B denotes the effective connection strength (*j*→*i*) between the populations. The coupling matrices are given by:


(5) $$\begin{array}{l} A=\left[\begin{array}{ccccccc} J_{\text{D}1,\text{D}1} & J_{\text{D}1,\text{D}2} & J_{\text{D}1,\text{FSI}} & J_{\text{D}1,\text{TAN}} & J_{\text{D}1,\text{TIN}} & 0 & 0\\ J_{\text{D}2,\text{D}1} & J_{\text{D}2,\text{D}2} & J_{\text{D}2,\text{FSI}} & J_{\text{D}2,\text{TAN}} & J_{\text{D}2,\text{TIN}} & 0 & 0\\ 0 & 0 & 0 & J_{\text{FSI},\text{TAN}} & J_{\text{FSI},\text{TIN}} & 0 & 0\\ 0 & J_{\text{TAN},\text{D}2} & 0 & J_{\text{TAN},\text{TAN}} & J_{\text{TAN},\text{TIN}} & J_{\text{TAN},\text{STN}} & 0\\ 0 & J_{\text{TIN},\text{D}2} & 0 & J_{\text{TIN},\text{TAN}} & J_{\text{TIN},\text{TIN}} & J_{\text{TIN},\text{STN}} & 0\\ 0 & 0 & 0 & J_{\text{STN},\text{TAN}} & J_{\text{STN},\text{TIN}} & 0 & 0\\ J_{\text{GPi},\text{D}1} & 0 & 0 & 0 & J_{\text{GPi},\text{TIN}} & J_{\text{GPi},\text{STN}} & 0 \end{array}\right]. \end{array}$$

(6) $$\begin{array}{l} B={\left[\begin{array}{c} J_{\text{D}1,\text{CTX}},J_{\text{D}2,\text{CTX}},J_{\text{FSI},\text{CTX}},0,0,J_{\text{STN},\text{CTX}},0 \end{array}\right]}^{T}. \end{array}$$

In the model, some of the projections were considered as a fixed parameter (listed in Table 2) for both the control and PD conditions, whereas others were assumed to be different in order to distinguish the control and PD conditions (listed in Table 3). These parameters were previously estimated (Bahuguna et al., 2017) based on animal experimental data (Connelly et al., 2010; Planert et al., 2010; Chuhma et al., 2011) used to tune mean-field network models of the control and parkinsonian BG.

**Table 2:** Connection strengths of the BG projections that are fixed for both the control and PD conditions (Bahuguna et al., 2017) and the corresponding transmission delays (Jaeger and Kita, 2011; Kita and Kita, 2011a).

**Projection**	**Strength (mVs)**	**Delay (ms)**
D1-MSN → D1-MSN	–0.69	0
D1-MSN → D2-MSN	–0.32	0
D1-MSN → GPi	–2.8	12
D2-MSN → D1-MSN	–1.15	0
D2-MSN → D2-MSN	–2.9	0
FSI → D1-MSN	–0.66	0
FSI → D2-MSN	–0.318	0
FSI → FSI	–0.0012	0
TIN → GPi	–0.78	1
STN → STN	0.05	0
STN → GPi	0.26	2

**Table 3:** Connection strengths of the BG projections that discriminate the control and PD states (Bahuguna et al., 2017) and the corresponding transmission delays (Jaeger and Kita, 2011; Kita and Kita, 2011a).

**Projection**	**Strength (mVs)**		**Delay (ms)**
	**Ctrl**	**PD**	
D2-MSN → TAN	–0.4	–2.1	7
D2-MSN → TIN	–0.45	–1.6	7
TAN → D1-MSN	–0.83	–0.93	1
TAN → D2-MSN	-1.2	–1.4	1
TAN → FSI	–1.6	–0.25	1
TAN → TAN	–0.6	–1.2	1
TAN → TIN	–0.27	–0.25	1
TAN → STN	–0.75	–0.4	1
TIN → D1-MSN	–0.3	–0.18	1
TIN → D2-MSN	–0.2	–0.6	1
TIN → FSI	–0.8	–1.5	1
TIN → TAN	–0.9	–0.5	1
TIN → TIN	–0.64	–0.03	1
TIN → STN	–2.0	–1.2	1
STN → TAN	1.7	1.4	2
STN → TIN	0.92	0.2	2

### 2.2 Cortical input

**Figure 2:**
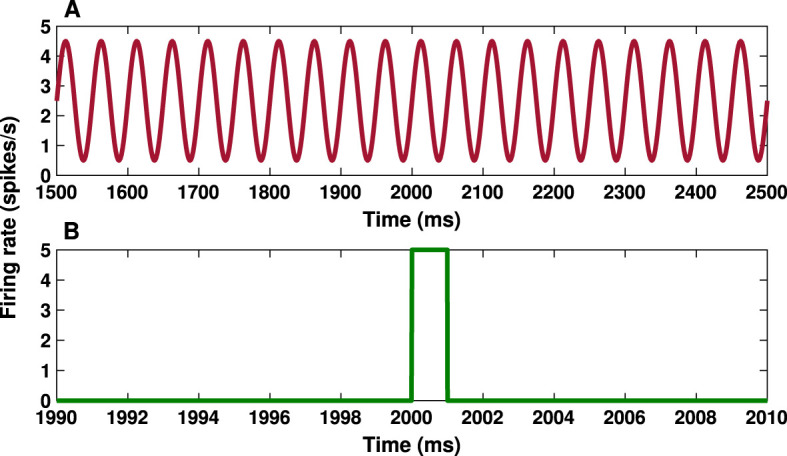
Cortical input and transient cortical stimuli. **(A)** Time course of the cortical input given by Equation (7) with amplitude *A* = 2 spikes/s and frequency *f* = 20 Hz. **(B)** Time trace of the transient cortical stimulation given by Equation (8) with amplitude *k* = 5 spikes/s and parameters *t*_*a*_ = 2, 000 ms and *t*_*b*_ = 2, 001 ms.

The cortical input (*I*_CTX_) in the model is considered as a feedforward excitatory input in the form of a sinusoidal current wave shown in Figure 2A (Bahuguna et al., 2017), targeting D1-MSN, D2-MSN, FSI and STN (also see Figure 1). The sinusoidal wave with amplitude *A* = 2 spikes/s and frequency *f* = 20 Hz acts as cortical input in the beta activation mode in the model (Mallet et al., 2005; Bahuguna et al., 2017):


(7) $$\begin{array}{l} I_{\text{CTX}}\left(t\right)=A\sin\omega t+C, \end{array}$$

where ω = 2π*f* is the angular frequency of the sinusoidal wave and *C* = 2.5 spikes/s.

Of note, *in vivo* neurophysiological recordings in control condition in animal models are typically carried out during two well-defined and controlled brain states, i.e., *cortical SWA* and *cortical beta activation* (Magill et al., 2006; Mallet et al., 2008a). The SWA state is dominated by low-frequency oscillations (1–2 Hz) and is qualitatively similar to activity observed during natural sleep, whereas the cortical activation mode contains patterns of activity that are more analogous to those observed during wakefulness, behaving state characterized by physiological levels of beta (13–30 Hz) oscillations. In PD condition, however, oscillatory activity in the STN-GPe network becomes excessively and selectively synchronized at beta frequencies in a brain state dependent manner after lesion of DA neurons in animal models (Mallet et al., 2008a).

Motivated by experimental data, previous modeling studies usually implemented cortical SWA or beta activation state by assuming a frequency-specific cortical drive to the BG (Ahn et al., 2016; Bahuguna et al., 2017). For instance, Ahn et al. (2016) used a *f* = 20 Hz cortical drive in their model of the parkinsonian BG. Instead, Bahuguna et al. (2017) modeled SWA state as a *f* = 2 Hz cortical input and beta activation state as a *f* = 20 Hz cortical input to construct the physiological (normal) as well as the pathological (parkinsonian) model. In their model, *f* = 20 Hz cortical input in the beta activation mode in the control condition did not produce abnormal beta rhythms and led to normal ranges of mean firing rates. Hence, *f* = 20 Hz cortical input in the beta activation mode in the PD condition does not simply transfer beta oscillations from the cortex to the BG since the same input in the control condition led to normal ranges of mean firing rates. Rather, here beta oscillations are generated due to parameter changes mentioned in Table 3. Accordingly, in this study, we used a *f* = 20 Hz cortical input to the BG to model both the control and PD networks in the cortical beta activation state.

### 2.3 Response to transient cortical stimuli

To test the response of the BG to transient cortical stimulation in both the control and PD conditions, we considered a short (with a duration of 1 ms) pulse stimulus (see Figure 2B). The cortical stimulation current is given by:


(8) $$\begin{array}{l} I_{\text{stim}}\left(t\right)=k\left[H\left(t-t_{a}\right)-H\left(t-t_{b}\right)\right], \end{array}$$

where *k* = 5 spikes/s is the amplitude of the stimulus pulse, δ*t* = *t*_*b*_−*t*_*a*_ = 1 ms determines the width of the stimulus pulse and *H*(*t*) is the Heaviside step function, i.e., *H*(*t*) = 1, if *t*≥0, and *H*(*t*) = 0, otherwise. The total time of the simulation was 3,000 ms and the stimulus onset time was at *t*_*a*_ = 2, 000 ms (see Figure 2B).

### 2.4 Transmission delays in the BG

The responses of downstream neural populations to motor cortex stimulation is typically characterized by an excitation-inhibition sequence with a specific latency (Kita and Kita, 2011a). In this study, we sought to investigate the dynamics of parkinsonian oscillations in the BG in the presence of transmission delays between different nuclei. These inter-population delays represent the latency of motor cortex stimulation-induced responses in the BG downstream networks (Nambu et al., 2000; Tachibana et al., 2008). The ranges of inter-population delays in the model were chosen based on previous experimental estimations in animal models (Jaeger and Kita, 2011; Kita and Kita, 2011a), which are given in Tables 2, 3. We also addressed the question that how changing transmission delays between different nuclei can alter population firing rates as well as oscillatory dynamics following cortical stimulation.

### 2.5 Power spectrum

The power spectrum of oscillatory signals was calculated by the fast Fourier transform (FFT) function implemented in MATLAB with a sampling frequency of *f*_*s*_ = 10 kHz.

## 3 Results

### 3.1 Firing rate modulation by transmission delays

Significant changes in the mean discharge rates and the pattern of neural oscillations in the CBGTC circuits following DA depletion are widely reported in experimental models of PD (Galvan et al., 2015; Asadi et al., 2022). While the discharge rate and pattern of the striatal neurons are under debate (Kita and Kita, 2011b; Valsky et al., 2020), increased firing rate of the STN cells and decreased firing rate of the GPe neurons have been verified in numerous experimental studies (Mallet et al., 2008b; Kita and Kita, 2011b).

**Figure 3:**
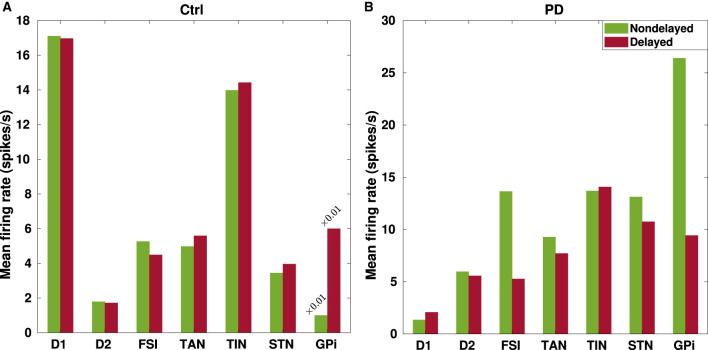
Changes in the mean firing rates of the BG nuclei due to transmission delays. Changes in the mean firing rates in the BG nuclei that were listed in Table 4 are visualized in the control **(A)** and PD **(B)** conditions for a better comparison between instantaneous (green) and delayed (red) BG network configurations.

The mean-field model used in this study is intrinsically identified by population firing rates. To check if the model output qualitatively mimics physiological estimates of firing rates in the BG, the mean firing rate of each nuclei is reported in Table 4 and the results are visualized in Figure 3 for a better comparison. The control and PD conditions were constructed by changing the coupling strengths between some of the BG nuclei, as indicated in Tables 2, 3, and the transmission delays were also included in the model. Together, these results confirm that the increasing/decreasing trend of the mean firing rate in the PD condition in comparison to the control condition in the model qualitatively agrees with experimental observations (Galvan et al., 2015; Asadi et al., 2022).

**Table 4:** Changes in the mean firing rates of different BG nuclei in the control and PD conditions when delay is introduced in the model based on values given in Tables 2, 3.

**Condition**		**Mean firing rate (spikes/s)**						
		**D1-MSN**	**D2-MSN**	**FSI**	**TAN**	**TIN**	**STN**	**GPi**
Ctrl	Nondelayed	17.1	1.79	5.26	4.97	13.98	3.44	0.01
		Delayed	16.96	1.73	4.49	5.59	14.42	3.96	0.06
PD	Nondelayed	1.34	5.96	13.64	9.27	13.69	13.11	26.39
		Delayed	2.07	5.56	5.26	7.7	14.07	10.74	9.42

For example, Figure 3 shows that the mean firing rate of D1-MSNs (D2-MSNs) is decreased (increased) in the PD condition with respect to the control condition whether or not delays are considered in the model. This is consistent with previous experimental observations that the mean firing rate of D1-MSNs is typically decreased (0.11 ± 0.04 Hz, PD vs. 1.61 ± 0.19 Hz, ctrl) (Ryan et al., 2018), whereas the mean firing rate of D2-MSNs is increased in the PD condition compared to the control condition in rodents (6.4 ± 2.7 Hz, PD vs. 2.1 ± 1.2 Hz, ctrl) (Kita and Kita, 2011b). Moreover, a study on parkinsonian rodents revealed that the firing rate of FSIs is increased in the PD condition compared to the control condition (Hernandez et al., 2013). Consistently, such an increase is successfully reproduced by our model as shown in Figure 3.

As it is validated in animal models of PD (Mallet et al., 2008a, 2012), the two main subtypes of GP neurons, i.e., GPe-TANs and GPe-TINs, can be identified by different firing rates and opposing phase relationships with STN neurons. As shown in Figure 3, distinct firing activity of the GPe subpopulations is reproduced in our model such that the mean firing rate of GPe-TAN is increased in the PD condition with respect to the control condition whether or not delays are present in the computational model. On the contrary, the mean firing rate of GPe-TIN is slightly decreased in the PD condition.

On the other hand, since the inhibitory input from the GPe to STN is suppressed following DA loss in PD, one expects that the firing rate of STN must be higher in the PD state compared to the normal condition. In fact, abnormally enhanced firing rate of the STN cells is a widely reported pathophysiological marker of PD (Brown et al., 2001; Hammond et al., 2007). The results in Figure 3 shows that the mean firing rate of the STN is increased in the PD condition compared to the control condition both in the nondelayed and delayed configurations in our model. This is also qualitatively consistent with experimental evidence on the increasing trend for the mean firing rate of STN in the PD condition, e.g., in parkinsonian rodents (17.1 ± 1.0 Hz, PD vs. 11.8 ± 0.70 Hz, ctrl) (Breit et al., 2007) as well as parkinsonian monkeys (25.8 ± 14.9 Hz, PD vs. 18.8 ± 10.3 Hz, ctrl) (Bergman et al., 1994). Furthermore, lower STN firing rates in the control condition are in agreement with *in vitro* and *in vivo* recordings in rats (i.e., 3.6 ± 1.0 Hz, ctrl) (Bevan and Wilson, 1999). Furthermore, as it is shown in Figure 3 the mean firing rate of GPi is significantly increased in the PD condition in comparison to the control condition which is in agreement with experimental findings in parkinsonian monkeys (77.9 ± 26.4, PD vs. 57.3 ± 17.3, ctrl) (Leblois et al., 2006). Taken together, abnormally enhanced mean firing rate of the BG output nucleus (i.e., GPi) increases the inhibitory drive to the thalamo-cortical circuits and, in this way, may contribute to the symptomatic expression of PD which is one of the widely accepted dynamical changes observed in PD.

**Figure 4:**
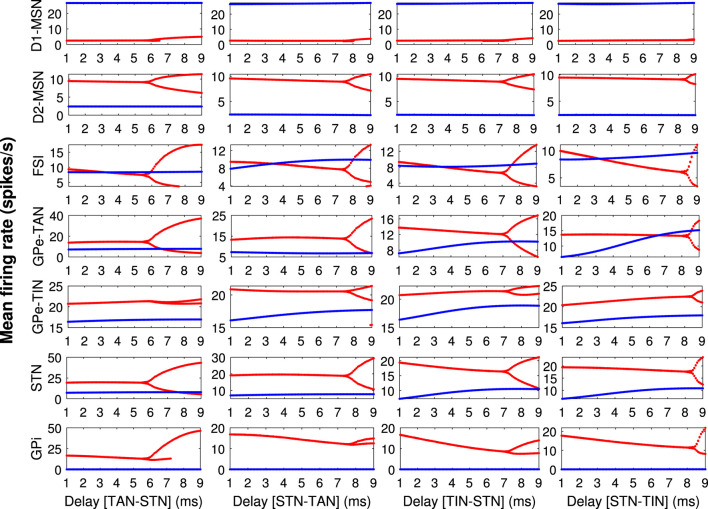
The bifurcation diagrams for different BG nuclei. The bifurcation diagrams for the mean firing rates of different BG nuclei (each row) including D1-MSN, D2-MSN, FSI, GPe-TAN, GPe-TIN, STN and GPi in the control (blue) and PD (red) conditions, when transmission delays in GPe-TAN ⇌ STN and GPe-TIN ⇌ STN pathways (each column) are varied. The bifurcation diagrams were obtained by calculating the minimum and maximum of the mean firing rates as a function of delays.

It was previously suggested that pathological beta oscillations in the PD condition may emerge due to bifurcation of the mean firing rates in the BG (Nevado-Holgado et al., 2011; Hu et al., 2022). To elucidate the relevance between inter-population transmission delays in our model and firing rate modulation in control (blue) and PD (red) conditions, the bifurcation diagrams for the mean firing rates of different BG nuclei including D1-MSN, D2-MSN, FSI, GPe-TAN, GPe-TIN, STN, and GPi are shown in Figure 4 for GPe-TAN ⇌ STN and GPe-TIN ⇌ STN delays. These bifurcation diagrams were obtained by calculating the minimum and maximum of the mean firing rates as a function of delays (Sun et al., 2023). Figure 4 indicates that in the control condition (blue) the minimum and maximum values of the mean firing rates in the BG overlap, but in the PD condition (red) bifurcation of the mean firing rates occurs, suggesting that parkinsonian oscillatory dynamics is more prone to modulation by transmission delays.

During the past years, several computational and experimental models emphasized on the role of the indirect pathway (i.e., cortex → D2-MSN → GPe ⇌ STN → GPi) and, specifically, the STN-GPe network in the emergence of pathological oscillations as well as alterations in the firing rates in the BG during parkinsonism (Bevan et al., 2002; Mallet et al., 2008a; Nevado-Holgado et al., 2010; Koelman and Lowery, 2019; Madadi Asl et al., 2022a). The STN-GPe network plays the role of a pacemaker for beta oscillation in the BG where its malfunction can spread the pathological dynamics in the entire CBGTC circuits (Brown et al., 2001; Mallet et al., 2008a). The GPe neurons may coordinate and propagate beta oscillations across the BG in a cell-type-specific manner (Mallet et al., 2008a, 2012). For instance, in-phase synchronization of GPe-TANs and STN neurons at beta frequencies likely reinforces abnormal beta oscillations in the STN-GPe network. Based on this background, in the current study we focus on the GPe-TAN → STN delay-dependent changes in the BG oscillatory activity.

**Figure 5:**
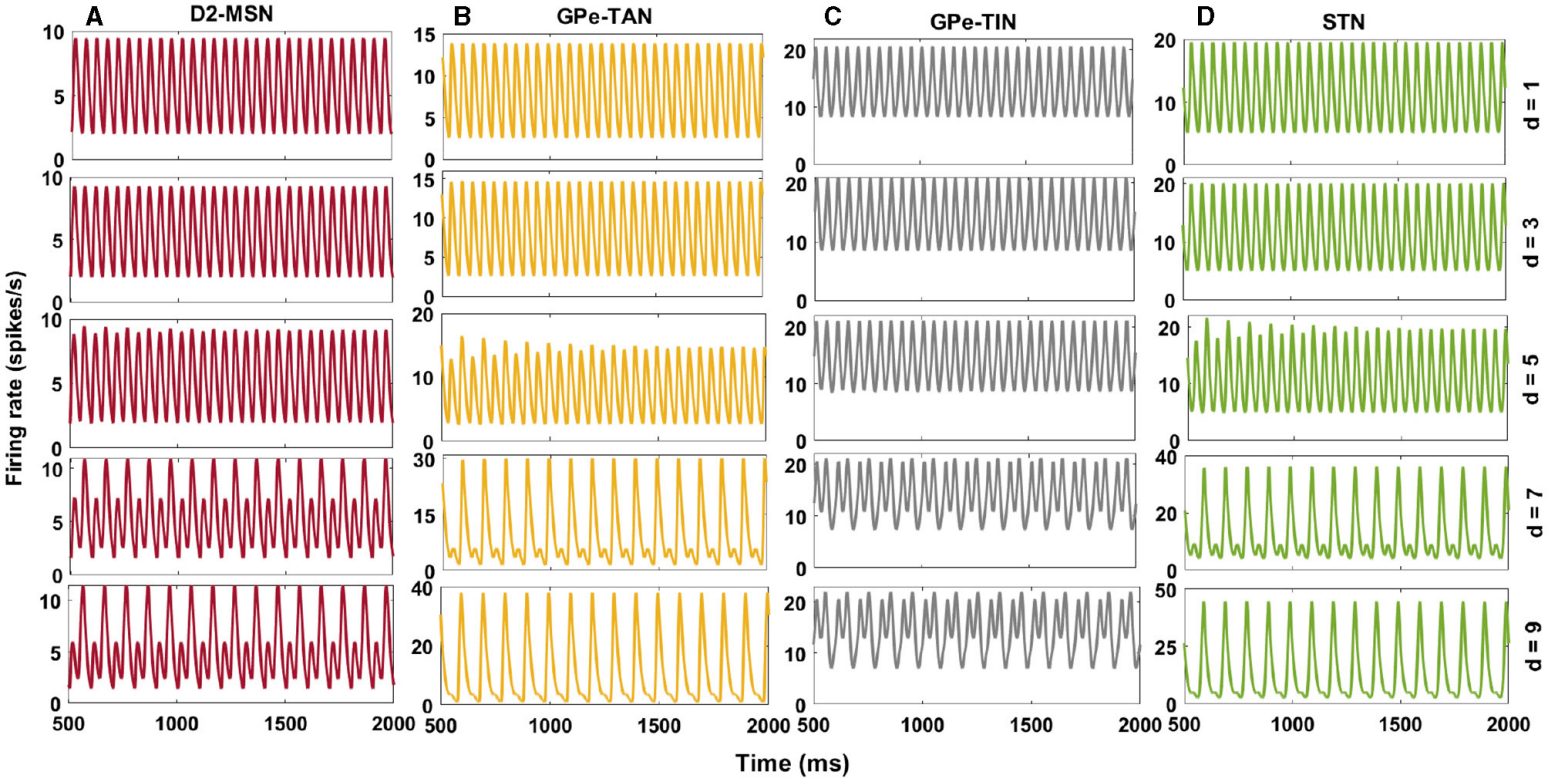
Delay-dependent changes in the firing rates of the BG nuclei in the PD condition. Firing rates of D2-MSN **(A)**, GPe-TAN **(B)**, GPe-TIN **(C)**, and STN **(D)** when the GPe-TAN → STN delay is varied from *d* = 1 ms (top) to *d* = 9 ms (bottom). Note that different vertical scales in subplots show different ranges for a better representation.

**Figure 6:**
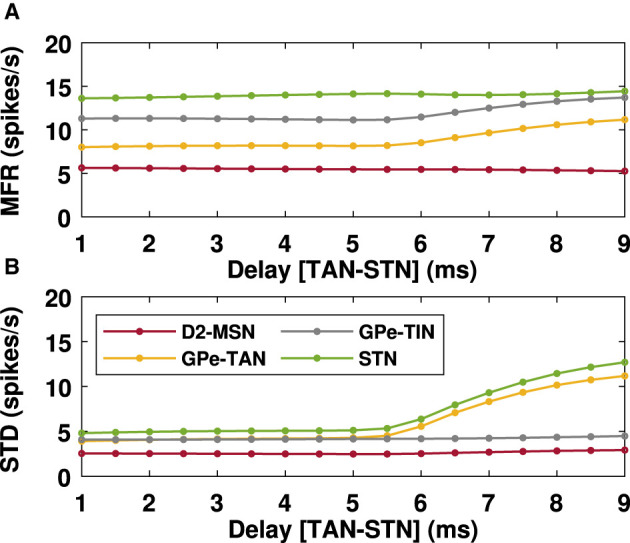
Mean firing rates of the BG nuclei in the PD condition in the presence of delays. Mean firing rates **(A)** of D2-MSN, GPe-TAN, GPe-TIN and STN when the GPe-TAN → STN transmission delay is varied from *d* = 1 ms to *d* = 9 ms (as in Figure 5). Standard deviations **(B)** are a measure of the amplitude of the oscillations of the mean firing rates.

To systematically investigate the effect of transmission delays on the oscillatory dynamics in the D2-MSN → GPe ⇌ STN pathway as the main part of the indirect pathway, we varied the GPe-TAN → STN transmission delay and recorded changes in the firing pattern (Figure 5) and mean firing rates (Figure 6) of the D2-MSN, GPe-TAN, GPe-TIN and STN. Together, these results suggest that while the firing patterns of the neural populations were altered by increasing the transmission delay (Figures 5A–D, top to bottom), their corresponding mean firing rates remained almost unchanged (Figures 6A, B). Interestingly, this observation is in agreement with experimental findings suggesting that changes of the BG dynamics during parkinsonism is accompanied by altered neural firing patterns (i.e., spiking, bursting and pausing properties) or firing regularity, rather than neural firing rates (Kita and Kita, 2011b; McConnell et al., 2012; Holt et al., 2019; Valsky et al., 2020). Figure 6 suggests that this phenomenon can be influenced by transmission delays between the BG nuclei in addition to changes in the connection strengths as it is usually considered.

### 3.2 Delay-dependent changes of oscillatory power

**Figure 7:**
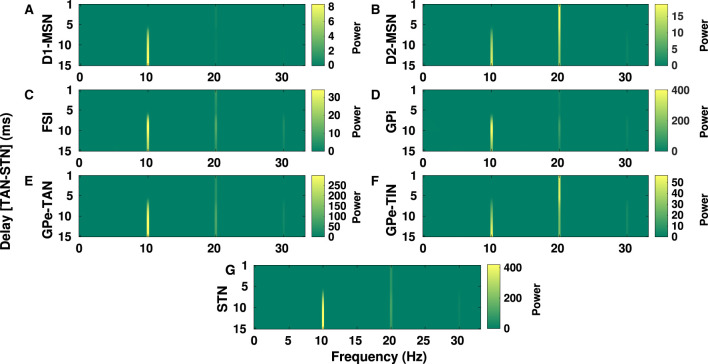
Delay-dependent changes in the power spectrum of BG oscillations in the PD condition. The color-coded power of oscillations in D1-MSN **(A)**, D2-MSN **(B)**, FSI **(C)**, GPi **(D)**, GPe-TAN **(E)**, GPe-TIN **(F)**, and STN **(G)** when the GPe-TAN → STN delay is varied from *d* = 1 ms to *d* = 15 ms. Note that different vertical scales in subplots show different ranges for a better representation.

**Figure 8:**
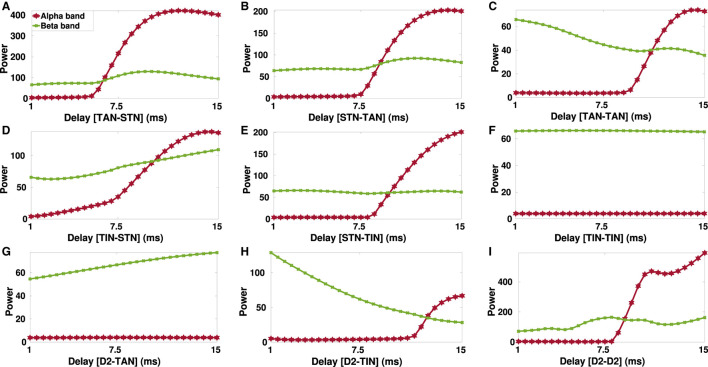
Delay-dependent changes in the power of alpha (8–13 Hz) and beta band (13–30 Hz) oscillations in the parkinsonian STN. The power of the STN alpha (red) and beta (green) oscillations when GPe-TAN → STN **(A)**, STN → GPe-TAN **(B)**, GPe-TAN → GPe-TAN **(C)**, GPe-TIN → STN **(D)**, STN → GPe-TIN **(E)**, GPe-TIN → GPe-TIN **(F)**, D2-MSN → GPe-TAN **(G)**, D2-MSN → GPe-TIN **(H)**, or D2-MSN → D2-MSN **(I)** delay is varied from *d* = 1 ms to *d* = 15 ms. Note that different vertical scales in subplots show different ranges for a better representation.

PD is typically characterized by excessive oscillatory activity in the BG at frequencies over alpha (8–13 Hz) and beta band (13–30 Hz) frequencies (Hammond et al., 2007; Kühn et al., 2009). Conditions required for generation of abnormal BG oscillations such as strengths of inter-population connections, imbalance of excitatory-inhibitory inputs and the role of transmission delays were previously addressed in a number of computational studies (Nevado-Holgado et al., 2010; Pavlides et al., 2012; Shouno et al., 2017). In Figures 7, 8 we have shown how changes in inter-population transmission delays in the BG could influence its oscillatory power.

In Figure 7, the GPe-TAN → STN transmission delay is varied from *d* = 1 ms to *d* = 15 ms and changes in the power spectrum of oscillations over alpha and beta frequencies are observed in different BG nuclei. Changes of transmission delays between other BG nuclei did not result in a significant change in the power spectrum of oscillations in the PD condition. The results presented in Figure 7 indicate that in all shown BG nuclei the power of alpha peak (~ 10 Hz) is increased when the GPe-TAN → STN transmission delay is increased from *d* = 1 ms to *d* = 15 ms. The beta peak (~ 20 Hz), however, showed no significant dependence on the GPe-TAN → STN delay in D1-MSN (A), FSI (C), GPi (D), GPe-TAN (E), and STN (G). Meanwhile, it was relatively decreased with increasing the GPe-TAN → STN transmission delay in the case of D2-MSN (B) and GPe-TIN (F).

To take a closer look at the changes of STN oscillations due to transmission delays in the PD condition, alpha and beta power spectra of the STN oscillatory activity are shown in Figure 8 when transmission delays between different nuclei in the D2-MSN → GPe ⇌ STN pathway are varied. Particularly, there seems to be a threshold transmission delay beyond which the STN oscillatory activity shows significant increase in the alpha frequency power (Figure 8, red curve), except for GPe-TIN → GPe-TIN (Figure 8F) and D2-MSN → GPe-TAN (Figure 8G) cases where alpha power was robust to the transmission delay changes.

On the other hand, the STN oscillatory power shows a descending trend when GPe-TAN → GPe-TAN (Figure 8C, green curve) and D2-MSN → GPe-TIN (Figure 8H, green curve) delays were increased from *d* = 1 ms to *d* = 15 ms. On the contrary, increasing delay in the GPe-TIN → STN (Figure 8D) and D2-MSN → GPe-TAN (Figure 8G) resulted in an ascending trend of the STN oscillatory power. Changes of delays in other pathways did not result in a significant change in the beta oscillatory power.

Together, these results suggest that at small transmission delays (i.e., <5 ms), the STN oscillatory activity is typically characterized by high-frequency oscillations in the beta frequency band (13–30 Hz), whereas at large delays (i.e., 8–15 ms), the STN oscillatory activity is mainly characterized by low-frequency oscillations in the alpha band (8–13 Hz). These findings suggest that in our model alpha oscillation emerges as a subharmonic of beta oscillation. As suggested by a number of experimental studies such a phenomenon may be disrupted in PD (Hughes and Crunelli, 2005; Lee et al., 2019).

### 3.3 Delay-dependent response to phase-specific stimuli

Abnormal neural oscillations in the parkinsonian BG are tightly related with abnormal STN-GPe dynamics (Bevan et al., 2002). However, experimental evidence highlighted the necessity of cortical inputs to the STN-GPe network for generating exaggerated beta band (12–30 Hz) oscillations during parkinsonism (Sharott et al., 2005; Mallet et al., 2006). As it was tested in computational models of parkinsonian BG, cortical excitatory input to the STN can either promote or suppress abnormal STN oscillations depending on their phase and strength (Shouno et al., 2017).

**Figure 9:**
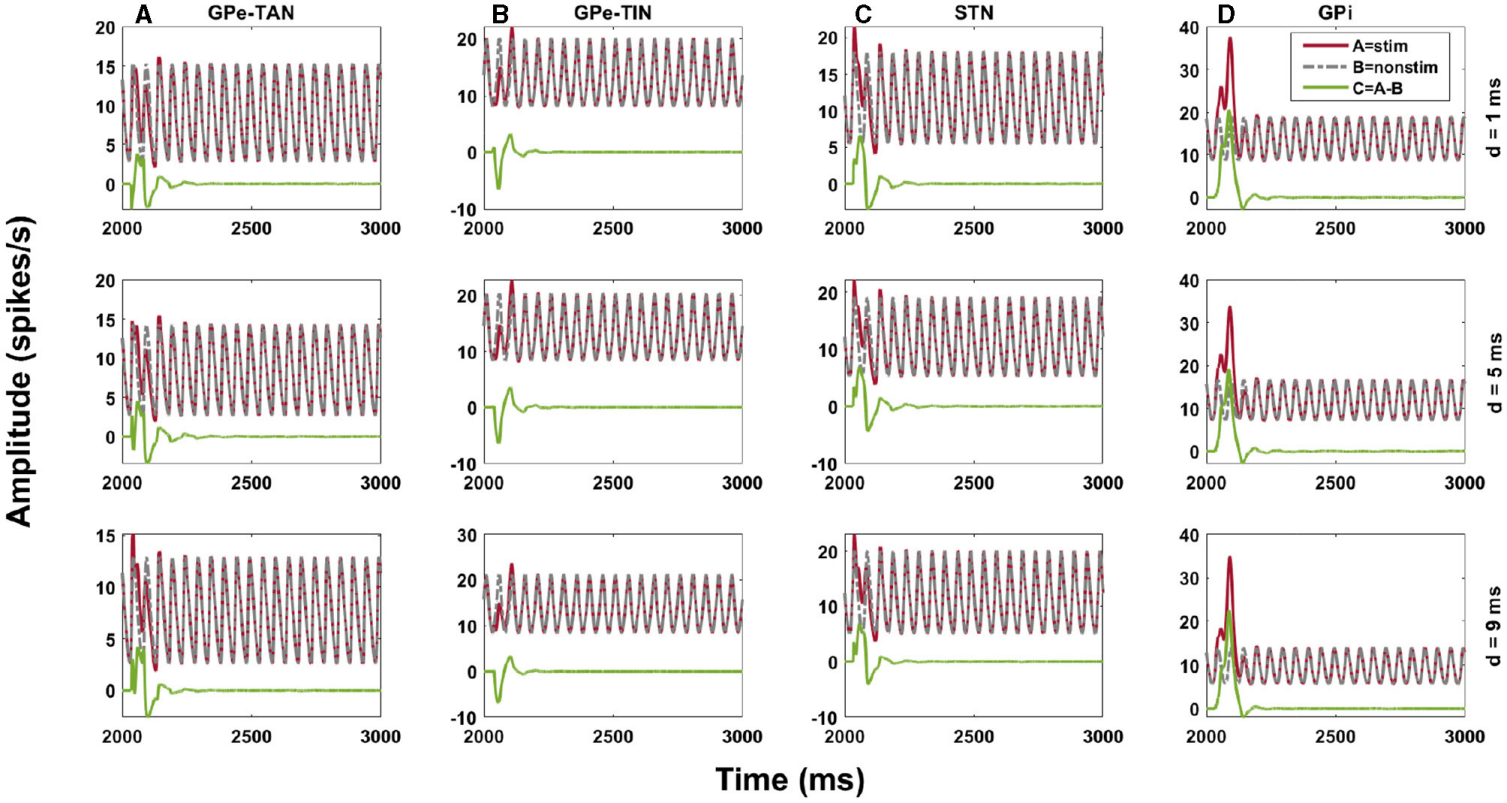
Amplitude response of the indirect pathway to transient cortical stimuli. Amplitude response curve (ARC) of the GPe-TAN **(A)**, GPe-TIN **(B)**, STN **(C)**, and GPi **(D)** for three representative delays in the D2-MSN → GPe-TAN pathway (each row), i.e., *d* = 1 ms, *d* = 5 ms and *d* = 9 ms in the control condition. The ARC (green) was defined as the difference between the stimulus-induced amplitude of oscillatory activity (red) and the none-stimulated setting (gray). Note that different vertical scales in subplots show different ranges for a better representation.

To shed light on delay-dependent changes of the BG response to cortical stimulation, we investigated how the transmission of transient cortical stimuli to the BG output nucleus (i.e., GPi) is mediated by delays in the indirect pathway, i.e., cortex → D2-MSN → GPe ⇌ STN → GPi pathway, both in the control and PD conditions. Specifically, our aim was to address the question that to what extent the BG oscillatory dynamics are robust against changing the phase of stimulus onset with respect to cortical oscillations. This was assessed by amplitude response curve (ARC) of each nuclei (Figure 9, green curve), i.e., the difference between the stimulus-induced amplitude of oscillatory activity (Figure 9, red curve) and the none-stimulated setting (Figure 9, gray curve). The cortical input, given by Equation (7), was modeled as an excitatory input in the form of a sinusoidal current, as shown in Figure 2A, and the stimulation, given by Equation (8), was considered as a short pulse stimulus (Figure 2B). Figure 9 shows examplary ARCs of the GPe-TAN, GPe-TIN, STN and GPi for three representative delays in the D2-MSN → GPe-TAN pathway (each row).

**Figure 10:**
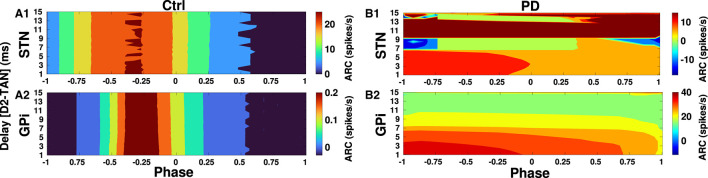
Delay-dependent amplitude response to transient phase-specific cortical stimuli. The color-coded ARC of the STN and GPi when the D2-MSN → GPe-TAN transmission delay and the phase of stimulation are systematically varied in the control **(A1, A2)** and PD **(B1, B2)** conditions. Note that different vertical scales in subplots show different ranges for a better representation.

**Figure 11:**
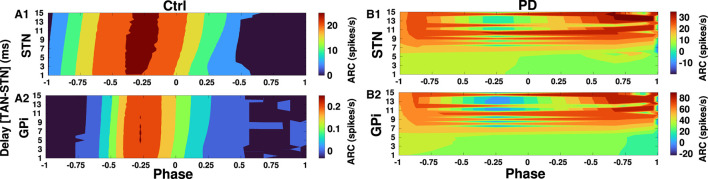
Delay-dependent amplitude response to transient phase-specific cortical stimuli. The color-coded ARC of the STN and GPi when the GPe-TAN → STN transmission delay and the phase of stimulation are systematically varied in the control **(A1, A2)** and PD **(B1, B2)** conditions. Note that different vertical scales in subplots show different ranges for a better representation.

Finally, in Figures 10, 11 we inspected delay-dependency and phase-specificity of the ARC of STN and GPi to transient cortical stimuli. To this end, we systematically varied D2-MSN → GPe-TAN delay (Figure 10) and GPe-TAN → STN delay (Figure 11) in the range *d* = [1, 15] ms as well as the onset phase of cortical stimulation with respect to cortical oscillatory activity in the range θ_stim_ = [−π, π] rad, and measured the ARC in each case both in the control and PD conditions. As shown in Figures 10A1, A2, 11A1, A2, in the control condition oscillatory dynamics of both the STN and GPi are robust against delays so that in a given stimulation phase (either delayed or advanced), the ARC remains fairly unchanged, i.e., the response of BG nuclei to cortical stimulation depends only on the phase of stimulation not on the delays. Note that in Figures 10, 11 the stimulation phase is rescaled to unity, i.e., θ_stim_ = [−1, 1].

However, as shown in Figure 10B1 in the PD condition the ARC amplitude of STN is maximized (red color) for small D2-MSN → GPe-TAN delays (i.e., <6 ms) and delayed stimulation phases (i.e., −π <θ_stim_ <0), and for large delays (i.e., >9 ms) typically irrespective of the stimulation phase. In the case of GPi, Figure 10B2 shows that in the PD condition the ARC amplitude is mainly maximized for small delays (i.e., <6 ms) and delayed stimulation phases (i.e., −π <θ_stim_ <0). The ARC is suppressed (green color) by increasing the D2-MSN → GPe-TAN delay and advancing the stimulation phase (i.e., 0 <θ_stim_ <π). In Figures 11B1, B2 in the PD condition, it can be observed that the ARCs of STN and GPi crucially depend on the GPe-TAN → STN delay such that for large delays (i.e., >8 ms) the ARC is typically enhanced (red color). Taken together, these results indicated that in the control condition the BG oscillatory dynamics is fairly robust against changes of inter-population transmission delays, whereas in the PD condition transmission delays may crucially support the emergence of abnormal rhythmogenesis in the BG.

## 4 Discussion

In this study, we employed a mean-field model of the BG and investigated the effect of inter-population transmission delays on the firing rate and power spectrum of the BG oscillations as well as the phase-specific amplitude response to transient cortical stimuli both in control and PD conditions. Our findings indicate that inter-population delays in the indirect pathway (i.e., cortex → striatal D2-MSN → GPe ⇌ STN → GPi) may alter the firing pattern of the BG oscillations but the corresponding mean firing rates are almost robust to delay changes. Moreover, delays in the indirect pathway may enhance the susceptibility of the BG oscillatory dynamics to display abnormal alpha and beta oscillatory activity in the PD condition in comparison to the control condition, suggesting that delays play an important role in abnormal rhythmogenesis in the parkinsonian BG.

The oscillations in response to transient cortical stimuli were previously attributed to the changes in the coupling strengths between the populations in similar mean-field models of the BG (Bahuguna et al., 2017). However, here we show that besides changes in the coupling strengths during transition from the control condition to the PD condition in the model, stimulation-induced oscillations are regulated by transmission delays between populations. In fact, delays crucially determine synchronization tendency of spiking neural networks by the modification of their phase response to stimuli (Madadi Asl and Ramezani Akbarabadi, 2023). By the same token, phase-specific response of the parkinsonian BG to transient cortical stimuli in our model is delay dependent. More specifically, in the control condition the BG oscillatory dynamics is robust against changes of delays, whereas in the PD condition delays and the phase of cortical stimulation shape the response of the BG nuclei to cortical stimulation. Significant amplitude response in the GPi, for example, can be achieved for small delays (i.e., <6 ms) in the indirect pathway and delayed phases of stimulation (i.e., −π <θ_stim_ <0) with respect to cortical oscillatory activity.

Movement impairment in PD is correlated with exaggerated beta oscillations in the cortex and STN (Levy et al., 2002; Mallet et al., 2008b). The reduction of abnormal beta oscillations by high-frequency (>100 Hz) deep brain stimulation (HF-DBS) positively correlates with improvement of PD motor symptoms (Meissner et al., 2005; Kühn et al., 2006, 2008). As shown computationally, beta oscillations can be modulated by phase-dependent administration of stimulation, where stimuli are time-locked to a certain phase of the ongoing beta oscillation (Weerasinghe et al., 2019; Duchet et al., 2020; West et al., 2022). For example, as shown computationally, precisely timed stimulation can recover physiological network states both in biologically inspired models of PD and essential tremor which reproduced the phase dependency of the response to phase-locked DBS (Duchet et al., 2020; West et al., 2022). The utility of such a strategy can be seen in suppressing pathological beta oscillations and controlling tremor, where stimulation is locked to a specific phase of the behavioral oscillation in patients (Cagnan et al., 2017; Holt et al., 2019). Our results shed light on phase-specificity of the response of the parkinsonian BG to stimulation and may contribute to a further understanding of phase relationships between the cortex and different BG nuclei in physiological and pathological states which paves the way toward developing novel and efficient phase-specific stimulation approaches.

A number of experimental studies suggested that PD is associated with exaggerated oscillatory activity in the BG at frequencies over alpha band (8–13 Hz) as well as beta band (13–30 Hz) (Levy et al., 2002; Stoffers et al., 2007; Kühn et al., 2009; Belova et al., 2023). For instance, STN field potential spectra recorded during rest from PD patients displayed inter-individual variability which can be categorized as those patients who show: (i) only high-frequency oscillations in the beta band (13–30 Hz), (ii) only low-frequency oscillations in the alpha band (8–13 Hz), and (iii) significant low- and high-frequency oscillations (Levy et al., 2002). Moreover, local field potentials (LFPs) recorded from inside the STN of PD patients during DBS surgeries revealed alpha-beta oscillatory peaks associated with disease duration, bradykinesia, and rigidity scores (Belova et al., 2023). This observation suggests that increased alpha-beta oscillations may emerge as additional phenomena complementing pathological beta oscillations in PD (Belova et al., 2023). Our model successfully reproduced such observations by incorporating realistic transmission delays (see Figures 7, 8). Delay-dependent changes in the power spectrum of parkinsonian oscillations suggest that patient-specific variability of delays in the BG (possibly due to nerve damage) may be a potential candidate to explain inter-individual variability in the STN field potential spectra of PD patients from a computational perspective.

Population models typically fail to capture the biological properties of realistic networks of neurons. Yet, mean-field models may be able to relate the microscopic-level neural dynamics to the macroscopic-level imaging measurements obtained in experimental studies (Di Volo et al., 2019; Lea-Carnall et al., 2023a,b). It has been shown that a mean-field model of conductance-based networks of spiking neurons with adaptation can accurately predict the spontaneous activity in asynchronous irregular regimes similar to *in vivo* activity as well as transient network response to external inputs (Di Volo et al., 2019). Here, we considered a simple mean-field firing rate model that merely represents the evolution of the mean firing activity in each BG population. Such a model inherently neglects the underlying dynamics and restricts information about the system, e.g., the structure of individual spike trains within a population. Although classical rate-based view of the functional structure of the BG was challenged by contradictory observations (Calabresi et al., 2014; Spix and Gittis, 2020), the advantage of simple models is that the limited parameter space minimizes the computational cost as opposed to more detailed simulation models such as spiking neural networks.

Another limitation of our model is that we tuned the strength of inter-population couplings to mimic parkinsonian oscillatory activity within the BG networks. Furthermore, cortical input was simplified as an external current. However, cortical input may shape abnormal rhythmic activity in the parkinsonian STN-GPe network (Mallet et al., 2006). During parkinsonism, beta band oscillatory activity of the cortex and STN are coherent and the beta band synchrony is significantly enhanced between the GPe and STN as well as between the STN and cortex (Sharott et al., 2005; Mallet et al., 2008b). As shown computationally, the excessive beta activity in the STN-GPe circuit is phase-locked to cortical beta input (Koelman and Lowery, 2019). Our model fails to capture the complex network interactions that give rise to pathological beta oscillations in PD, but still can make qualitative predictions about the potential role of transmission delays in the normal and parkinsonian BG dynamics.

Furthermore, for the sake of simplicity, we limited our analysis to the STN-GPe oscillatory dynamics and BG output. Consequently, due to lack of knowledge other nuclei such as thalamus as well as other projections such as thalamo-striatal and striatal cholinergic interneuron inputs were not considered in the model. Although there is limited information about the thalamo-striatal system and the regulatory effects of dopamine on thalamic transmission compared to the cortico-striatal system, these inputs may play a role in the PD pathophysiology (Smith et al., 2004, 2014; McCarthy et al., 2011; Kondabolu et al., 2016). For instance, besides the cortico-striatal projections, the MSNs are also targeted by the thalamo-striatal projections. These thalamic inputs may modulate cortico-striatal transmission via regulation of striatal cholinergic interneurons, possibly contributing to some symptoms of PD (Smith et al., 2011). Furthermore, as shown experimentally, activation of striatal cholinergic interneurons can generate beta oscillations in cortical-striatal circuits, leading to parkinsonian-like motor deficits in animal models (Kondabolu et al., 2016).

Here, we investigated differences in responses of normal and PD model states to a wide range of transmission delays within the BG. These transmission delays can be attributed to potential inter-individual delay variations from subject to subject due to anatomical and neurophysiological variability in brains across patients, which may contribute to inter-individual variations in the clinical expression of PD. For instance, experimentally observed differences in power spectral densities in different PD patients may be related to such anatomical and neurophysiological differences from one patient to another (Levy et al., 2002). Yet, future experiments must be devoted to measure transmission delays within and between BG nuclei to determine whether these delays are changed with disease progression which seems crucial for understanding the mechanisms behind oscillatory dynamics in the BG.

Finally, synaptic connections in brain circuits as well as other biological systems are constantly modified by plasticity mechanisms (Burke and Barnes, 2006; Madadi Asl and Ramezani Akbarabadi, 2021). In our model, however, the connection strengths were assumed to be static, i.e., they do not dynamically evolve. Taking into account the effect of plasticity in future studies allows for a more accurate inspection of maladaptive/compensatory patterns of activity and connectivity shaped by plasticity mechanisms during parkinsonism (Madadi Asl et al., 2022b). For instance, spike-timing-dependent plasticity (STDP) (Gerstner et al., 1996; Markram et al., 1997; Bi and Poo, 1998), a temporally precise model for synaptic plasticity to modify the synaptic strengths based on correlated neural firings, promotes strong connections between correlated neurons and suppresses synapses between uncorrelated neurons in a computational PD model (Madadi Asl et al., 2022a). Therefore, activity of neurons shapes the overall connectivity pattern which, in turn, modulates the firing activity (Aoki and Aoyagi, 2009; Madadi Asl et al., 2018c; Madadi Asl and Ramezani Akbarabadi, 2023).

In this context, functional consequences of synaptic plasticity such as phase synchronization of oscillations critically depend on the transmission delays in neural interactions (Lubenov and Siapas, 2008; Madadi Asl et al., 2017, 2018a). For example, it has been shown that STDP combined with delayed neural interactions can lead to the emergence of multistable states in a computational model of PD (Madadi Asl et al., 2022a), i.e., physiological states (weak synchrony, weak connectivity) as opposed to pathological states (strong synchrony, strong connectivity). Future studies should incorporate phase-dependent plasticity rules (Seliger et al., 2002; Aoki and Aoyagi, 2009) in large adaptive networks described by mean-field approximations (Duchet et al., 2022) to investigate how regimes of delay-dependent response to phase-specific stimuli (Madadi Asl et al., 2023) as well as neural firing and spectral properties are modified by plasticity mechanisms. Such a prospect may provide a useful framework for the development of new therapeutic approaches aimed at shifting the brain dynamics from pathological states to more physiologically favored states.

## Data availability statement

The original contributions presented in the study are included in the article/supplementary material, further inquiries can be directed to the corresponding author.

## Author contributions

AA: Formal analysis, Investigation, Methodology, Visualization, Writing – review & editing. MM: Conceptualization, Formal analysis, Methodology, Project administration, Visualization, Writing – original draft, Writing – review & editing. AV: Conceptualization, Formal analysis, Methodology, Supervision, Writing – review & editing. MP: Conceptualization, Formal analysis, Funding acquisition, Methodology, Supervision, Writing – review & editing.
